# Using Open-Source Intelligence to Detect Early Signals of COVID-19 in China: Descriptive Study

**DOI:** 10.2196/18939

**Published:** 2020-09-18

**Authors:** Elizabeth Benedict Kpozehouen, Xin Chen, Mengyao Zhu, C Raina Macintyre

**Affiliations:** 1 Biosecurity Program The Kirby Institute for Infection and Immunity University of New South Wales Sydney Australia; 2 School of Public Health and Community Medicine University of New South Wales Sydney Australia

**Keywords:** COVID-19, infectious disease, surveillance, epidemiology, biosecurity

## Abstract

**Background:**

The coronavirus disease (COVID-19) outbreak in China was first reported to the World Health Organization (WHO) on December 31, 2019, and the first cases were officially identified around December 8, 2019. Although the origin of COVID-19 has not been confirmed, approximately half of the early cases were linked to a seafood market in Wuhan. However, the first two documented patients did not visit the seafood market. News reports, social media, and informal sources may provide information about outbreaks prior to formal notification.

**Objective:**

The aim of this study was to identify early signals of pneumonia or severe acute respiratory illness (SARI) in China prior to official recognition of the COVID-19 outbreak in December 2019 using open-source data.

**Methods:**

To capture early reports, we searched an open source epidemic observatory, EpiWatch, for SARI or pneumonia-related illnesses in China from October 1, 2019. The searches were conducted using Google and the Chinese search engine Baidu.

**Results:**

There was an increase in reports following the official notification of COVID-19 to the WHO on December 31, 2019, and a report that appeared on December 26, 2019 was retracted. A report of severe pneumonia on November 22, 2019, in Xiangyang was identified, and a potential index patient was retrospectively identified on November 17.

**Conclusions:**

The lack of reports of SARI outbreaks prior to December 31, 2019, with a retracted report on December 26, suggests media censorship, given that formal reports indicate that cases began appearing on December 8. However, the findings also support a relatively recent origin of COVID-19 in November 2019. The case reported on November 22 was transferred to Wuhan approximately one incubation period before the first identified cases on December 8; this case should be further investigated, as only half of the early cases were exposed to the seafood market in Wuhan. Another case of COVID-19 has since been retrospectively identified in Hubei on November 17, 2019, suggesting that the infection was present prior to December.

## Introduction

### Background

Severe acute respiratory syndrome coronavirus 2 (SARS-CoV-2) is a new betacoronavirus that was first reported in Wuhan, China, in December 2019; this virus has caused the worst pandemic of the past 100 years [1-3]. On December 31, 2019, Chinese authorities notified the World Health Organization (WHO) of an outbreak of pneumonia in Wuhan [2]. The WHO declared the coronavirus disease (COVID-19) outbreak to be a public health emergency of international concern on January 30, 2020, and it was declared a pandemic on March 12 [2,4]. It is commonly believed that the outbreak began in early December 2019.

Coronaviruses are a large family of viruses that are found in many different species of animals, including camels, cattle, cats, and bats. Zoonotic coronaviruses that have emerged in humans are Middle Eastern respiratory syndrome coronavirus (MERS-CoV), sudden acute respiratory syndrome coronavirus (SARS-CoV), and now SARS-CoV-2. This is the third time in two decades that a zoonotic coronavirus has emerged from animals to infect humans [4]. Of the betacoronaviruses, SARS-CoV-2 is more closely related to SARS-CoV than to MERS-CoV [5].

The origin of SARS-CoV-2, its intermediary animal host, and the mechanism of its species jump to humans are not known [6,7]. Initially, it was believed that the COVID-19 pandemic originated at the Huanan Seafood Wholesale Market located in Wuhan, China, where farm animals, bats, and snakes were also sold [8]; this is still believed by many people. Approximately half of the initial cases were exposed to the seafood market; however, the first two identified cases did not visit the seafood market [9]. Viral RNA was found in environmental samples from the wet market, such as surfaces [10]. Phylogenetic analysis revealed that the viral RNA found in the environmental samples was very closely related to viruses sampled from the earliest Wuhan patients, suggesting that the market played a role in the early spread of the virus [10]. The source of positive environmental samples from the market is unknown, and animal samples from the market are not available. Therefore, it has not been possible to identify an animal source at the market [10].

On March 2020, however, the timeline of the pandemic was questioned when it was determined that the first person infected with the new disease may have been a Hubei resident who was infected on November 17, 2019 [11]. However, official information states that the first patient presented on December 8, 2019, and that the first exposure may have been around December 1 in the Huanan Seafood Wholesale Market [2]. Local health authorities initially failed to report the coronavirus epidemic, resulting in a delay in reporting it to the WHO until December 31, 2019.

Emerging infectious diseases are becoming increasingly common [12,13]. The world is increasingly interconnected; therefore, it is essential to identify epidemics early [14]. A disease with true epidemic potential can grow exponentially within weeks or months; thus, each day of delay is a lost opportunity for prevention [15]. Rapid prediction, detection, and surveillance of outbreaks are critical in fighting emerging infectious diseases with epidemic potential [12]. The media may have reported a surge of unknown or undiagnosed cases of severe pneumonia, severe acute respiratory illness (SARI), or other related diseases prior to the official reporting of confirmed COVID-19 confirmed cases in China. Epidemic intelligence from open-source, informal data can provide early warnings of public health emergencies [12-14,16,17].

EpiWatch is a curated epidemic observatory that searches media reports, press releases, official reports, and social media for early detection of outbreaks of infectious diseases; it can be tailored for different languages [18]. EpiWatch provides early outbreak alerts and can be used to detect and monitor early reports of potential COVID-19 outbreaks through publicly available sources in settings with poor disease surveillance or censorship of information [19]. Early reports of unknown pneumonia in Hubei Province in China that appeared prior to official reports can be identified using open-source data, providing insight into whether COVID-19 was present in China before December 2019.

### Aim

The aim of our study was to use open-source data to identify early signals of pneumonia and SARI in China prior to official recognition of the COVID-19 outbreak in December 2019.

## Methods

EpiWatch is an open-source epidemic observatory that was developed at the University of New South Wales as a management web application enhanced by machine learning; it has been used to collect outbreak data since 2016. The principle of EpiWatch is that cases of infectious diseases or outbreaks may be reported in the news or discussed on social media before official notification by health authorities. EpiWatch mines open-source data to detect early signals, which can be customized for common clinical infectious disease syndromes. Many countries have weak or delayed surveillance systems and poor reporting. In other countries, censorship may prevent notification of serious epidemics. Open-source data can be used to help identify epidemic signals in such circumstances.

The system includes three major features. First, reports are gathered from international organizations and news outlets by an intelligent and modular system. An administrator can easily add new sources without requiring further development of the application. The data collected include news reports and social media posts as well as grey literature, such as government reports. If the format in which data is delivered changes for a given source, an administrator can promptly modify the system to adapt to this change. This includes adding or changing the languages used for searching. The system is set up to support a variety of intelligent data gathering elements, such as natural language processing algorithms, regular expression matching, and supervised machine learning algorithms, to process reports and attempt to identify important data points such as outcomes, locations, and diseases mentioned within the gathered data.

Second, EpiWatch reports are reviewed by a team of epidemiologists, ensuring a good level of quality control as well as increased accuracy and relevance. The EpiWatch management system is a web application that enables the internal team to log on and review reports and key data points identified by the automated data gathering system. The team can check the data collected by the automated system and correct any mistakes that are present. A machine learning system learns from this human input and corrections and uses that feedback to improve its ability to group reports and identify key information over time.

Finally, the EpiWatch management web application consists of two software programs. One is a web application that is built on the Vue framework, and the other is a server-side application built on the NodeJS framework. Both applications are written in JavaScript. The third software program is the data-gathering program, which is also a NodeJS application written in JavaScript. This program is scheduled to run on a regular basis to re-scan sources at intervals chosen by the system administrator. Searches can be tailored for specific languages or regions as well as for specific infectious disease syndromes. The data are stored in a PostgreSQL database. Most of the data is textual in nature and is easily compressed; therefore, the storage requirements are currently very modest (<100 MB). The EpiWatch observatory is managed and funded by the Australian National Health and Medical Research Council (NHMRC) Centre of Research Excellence, Integrated Systems for Epidemic Response (ISER) and is managed by staff at the Biosecurity Program, The Kirby Institute, University of New South Wales Sydney.

To capture early reports of SARI or pneumonia-related illnesses in China, searches were performed in the Chinese language using keywords reflecting severe acute respiratory syndrome or pneumonia as well as Wuhan and China as geolocations. We performed searches from October 1, 2019, to February 14, 2020. Any relevant news reports with the keywords *pneumonia*, *SARI* and related terms, and *coronavirus* were extracted. The information before December 31, 2019 (the date on which the WHO was notified of the COVID-19 outbreak) was reviewed for potential early signals of COVID-19. Google and the Chinese search engine Baidu were used [20,21]. Reports in Chinese were retrieved and reviewed by EK, XC, and MZ and translated to English.

## Results

**Figure 1:**
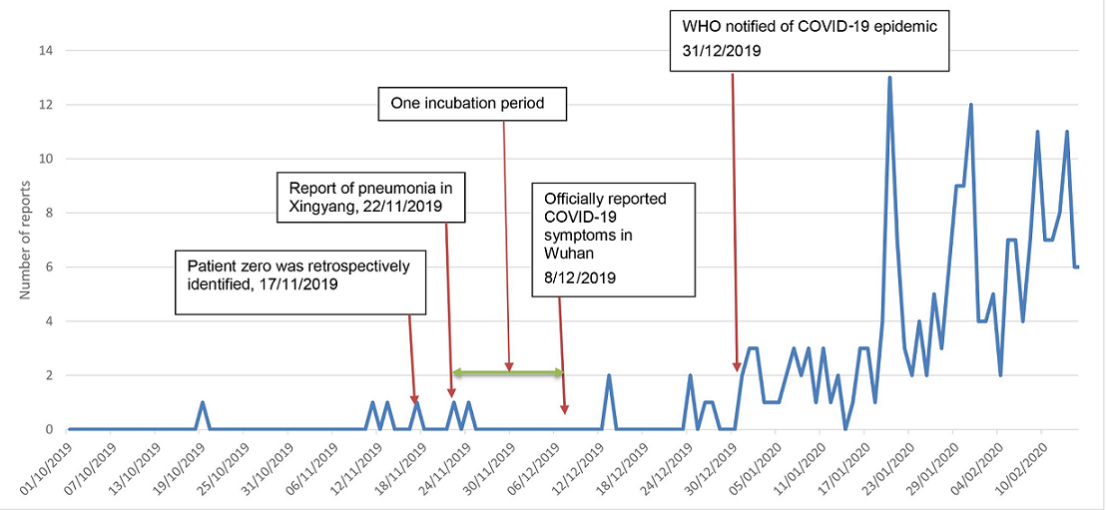
Reports of pneumonia, severe acute respiratory illness, or coronavirus from October 1, 2019, to February 14, 2020. COVID-19: coronavirus disease; WHO: World Health Organization.

Between October 2019 and February 2020, a total of 218 reports were found and included in the study. There were no duplicates. We identified two potentially relevant news reports prior to December 31, 2019. Figure 1 shows the number of pneumonia and/or SARI reports from October 1, 2019, to February 14, 2020. It shows an increase in reports after the official notification to the WHO on December 31, 2019. A report appeared on December 26, 2019, with the heading “One sample is suspected as novel coronavirus”; this report appears to have been retracted, as the link to the news item has become invalid [22]. We found 11 reports of cases of pneumonia between October 1 and December 31, 2019, including a case identified retrospectively in March 2020, which is believed to be an index case. The number of reports in the same period one year prior was determined for comparison; there were 12 reports in 2018. Of the 11 reports in 2019, 3 (27%) were cases of pneumonia of unknown cause, and 7 (64%) had known causes; 2 reports (18%) were related to pulmonary nodules, 3 (27%) were caused by lung cancer, cerebral infarction, or asthma, and 2 cases (18%) were caused by bacterial infection. The information of interest was the single report of unknown serious pneumonia in November 2019 in Hubei, the province in China where the COVID-19 pandemic arose.

**Figure 2:**
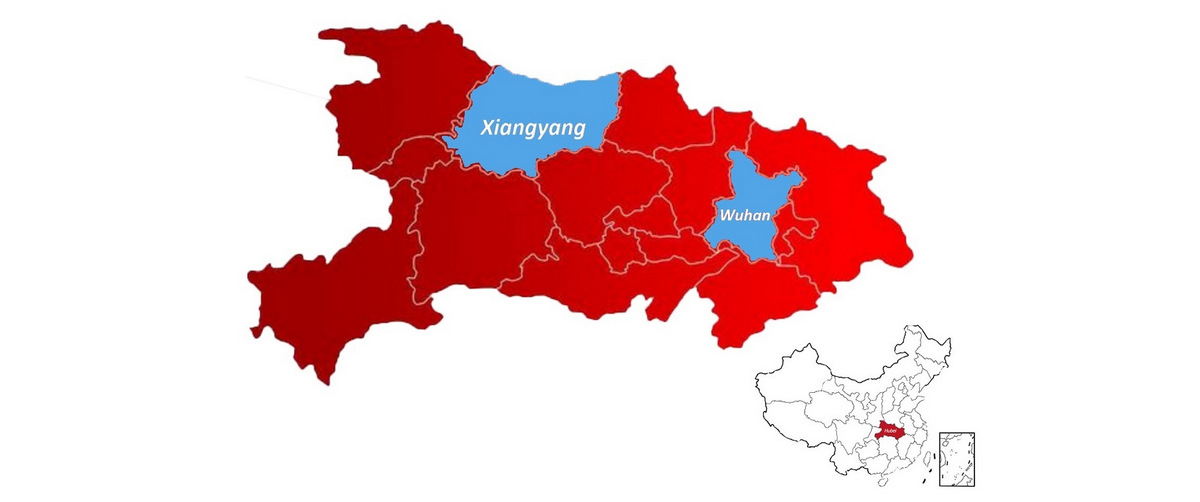
Location of Xiangyang relative to Wuhan within Hubei Province.

On November 22, 2019, a local newspaper, the *Wuhan Evening News*, reported that a patient with severe pneumonia of unknown cause was taken to Wuhan as an emergency transfer by helicopter from Xiangyang in Hubei Province, 325 kilometers from Wuhan [23]. Figure 2 shows the location of Xiangyang in relation to Wuhan. After November 22, there were no reports of pneumonia in the local media, although it was later confirmed that by December 30, 2019, there were 27 cases of pneumonia of unknown cause in Wuhan.

## Discussion

The origin of the COVID-19 epidemic is unknown. Only half the initial patients were exposed to the Huanan Seafood Wholesale Market [9], and the first two cases in Wuhan did not visit the market. No pneumonia or SARI signals in Wuhan were identified prior to December 31, which supports the relatively recent emergence of COVID-19. However, open-source intelligence identified a case of severe pneumonia in Xiangyang, Hubei Province, 325 km from Wuhan, who was transferred to Wuhan for treatment on November 21, 2019. This case may be part of an early outbreak cluster. In early March, it was reported that the first case of COVID-19, a different case identified retrospectively, may have been observed on November 17 [24-26]. Approximately one COVID-19 incubation period (2 weeks) [9] after November 17 to November 21, the first formally reported cases in Wuhan became symptomatic (around December 1-8). If no definitive diagnosis was made, further diagnostic investigation of the case from Xiangyang and epidemiological investigation is warranted to determine if this case did have COVID-19. There may be a connection between the Xiangyang patient and an unidentified early cluster of COVID-19.

From December 31, 2019, through January 3, 2020, a total of 44 case patients with pneumonia of unknown etiology were detected by syndromic surveillance by the China Center for Disease Control and Prevention. Exposure to the Huanan Seafood Wholesale Market was initially suspected to be the origin of the virus, and the market was closed on January 1, 2020. At least 35 environmental samples from the seafood section of the Huanan Seafood Market in Wuhan tested positive for the virus [27,28]. However, the first two cases did not report visiting the seafood market, and there is no epidemiological link between the first patient and later cases [5,28]. This, together with the identification of at least two severe pneumonia cases in November (the one identified in this study and the case on November 17, 2019), suggests that the epidemic originated earlier than December 2019.

The absence of news reports in December is curious given that the outbreak appears to have been recognized in early December. It is possible that media reporting was censored; this is supported by what appears to be a retracted news item on December 26. The findings also support the relatively recent origin of COVID-19 in November 2019. The case reported on November 22 was transferred to Wuhan approximately one incubation period before the first cases were reported on December 8. This case should be further investigated, as only half of the early cases were exposed to the Huanan Seafood Market. The Chinese government has been questioned about its failure to identify and report the epidemic early, which resulted in worldwide spread of the disease and led to a pandemic [29]. Surveillance of waste water may also shed light on the origin. A sample of stored waste water in Spain tested positive for SARS-CoV-2 in March 2019, raising questions about whether the infection was present much earlier than December that year [30].

Epidemic diseases grow exponentially and rapidly [31], as seen in China, Europe, and the United States [32]. Early detection and epidemic control can reduce epidemic growth and prevent further spread. Open-source intelligence is a potential tool to aid early detection, especially where formal surveillance data are lacking. Although these data are not validated, once a signal is detected, it can and should be formally investigated, tested, and validated. The use of open-source epidemic intelligence can supplement conventional surveillance to provide early detection of serious emerging epidemics, especially where official disease surveillance reporting is lacking.

## Abbreviations

COVID-19: coronavirus disease
ISER: Integrated Systems for Epidemic Response
MERS-CoV: Middle Eastern respiratory syndrome coronavirus
NHMRC: National Health and Medical Research Council
SARI: severe acute respiratory illness
SARS-CoV: severe acute respiratory syndrome coronavirus
SARS-CoV-2: severe acute respiratory syndrome coronavirus 2
WHO: World Health Organization
